# Cochlear Implantation Following Neonatal Meningitis: A Rare Case Report

**DOI:** 10.1155/crot/6647034

**Published:** 2026-05-19

**Authors:** Dagmar Hošnová, Milan Urík, Jan Šíma, Vít Kruntorád

**Affiliations:** ^1^ Department of Pediatric Otorhinolaryngology, University Hospital Brno, Černopolní 9, Brno, 61300, Czech Republic, fnbrno.cz; ^2^ Faculty of Medicine of Masaryk University in Brno, Kamenice 5, Brno, 62500, Czech Republic

**Keywords:** cochlear implantation, neonatal meningitis, sensorineural hearing loss

## Abstract

Bacterial meningitis in neonates may lead to profound sensorineural hearing loss and rapid postinflammatory cochlear changes that complicate auditory rehabilitation. We present two infants with bilateral profound hearing loss following neonatal bacterial meningitis who underwent early bilateral cochlear implantation. Radiological findings were limited or subtle, but intraoperative findings confirmed cochlear obstruction/ossification requiring adapted surgical management. Full electrode insertion was achieved in both children. Postoperative outcomes showed auditory benefit in both cases, although further development was strongly influenced by associated neurological comorbidities. These cases highlight the importance of early hearing assessment, repeated audiological follow‐up, prompt imaging, and timely cochlear implantation in postmeningitic hearing loss.

## 1. Introduction

The incidence of bacterial meningitis in developed countries is estimated to be 2–5 per 1000 inhabitants, and in developing countries that figure is as much as 10 times higher. Neonatal meningitis, with an incidence of 0.3 per 1000 inhabitants, poses extreme risk to an immature organism. It is a life‐threatening disease with high morbidity and severe neurological consequences. Neonatal meningitis is defined as meningitis occurring within the first 28 days of life [1, 2].

Thanks to the implementation of antibiotic prophylaxis against Group B streptococci, the number of early‐onset meningitis cases has decreased in developed countries. Nevertheless, *Streptococcus agalactiae* remains the most common pathogen, followed by *Escherichia coli*. The risk of infection increases with lower gestational age. Due to immaturity of the immune system, a wide range of neurological complications can occur [3, 4].

Hearing loss is one of the severe consequences of bacterial meningitis. Because cochlear ossification can begin just a few weeks after disease onset, early diagnosis is crucial and should be followed by timely cochlear implantation.

By means of newborn hearing screening, we can detect severe hearing loss by 3 months of age. Every child with profound hearing impairment initially undergoes hearing aid correction for a period of 3–6 months. In most cases, however, this correction is insufficient, and, if the effect is inadequate, we proceed with cochlear implantation at an early stage. The ideal age for implantation is 12 months, as the auditory pathway is sufficiently mature at this point. By this age, we expect no further natural hearing improvement, and we can reliably determine through behavioral methods whether the child would benefit from hearing aids.

In a case of bacterial meningitis, the situation is entirely different. Labyrinthitis, which causes profound hearing loss, is followed by fibrosis and ossification, which can prevent insertion of the electrode array. This process necessitates a more aggressive and rapid approach. Hearing aids are not indicated in such cases and, despite low body weight and gestational age, we proceed with cochlear implantation as quickly as possible [4].

Here, we present two infants who developed profound hearing loss after neonatal bacterial meningitis and underwent bilateral cochlear implantation at a very early age. These cases illustrate the diagnostic and surgical challenges of urgent cochlear implantation in this setting.

## 2. Case Report

This report describes the clinical course of two infants from the onset of meningitis through hearing assessment, cochlear implantation, and postoperative follow‐up. To provide institutional context for the rarity of this indication, we also summarize all neonates with bacterial meningitis evaluated at our tertiary center between 2015 and 2024 (Table 1). However, the present report focuses specifically on the two children who developed profound sensorineural hearing loss requiring cochlear implantation.

**TABLE 1:** Clinical characteristics of neonates with bacterial meningitis evaluated at our center.

	Preterm (32–36 tg)	Term (37–41 tg)
Number of newborns	4	12
Weight g	1805 (1450–2570)	3295 (2560–3880)
Early onset	0	2
Late onset	4	10
Postnatal age at diagnosis (days)	16	14
*Str.* agal	2	9
*E.* coli	1	2
Others	1 *(Serratia marcescens)*	1 *(Chryseobacterium)*
SNHL	2	0
Neurological deficits	2	2

Among the 16 neonates, 4 were born preterm and 12 at term. *Streptococcus agalactiae* was the most common pathogen. Severe sensorineural hearing loss occurred in two children, both from the preterm subgroup. Neurological complications were observed in four patients.

### 2.1. Case 1

A 1.5‐year‐old girl was first examined at our clinic at 3 months of age following bacterial meningitis. She was born from a multiple pregnancy (biamniotic and bichorionic), with fetal reduction from twins to a singleton performed at the parents’ request in the 8th week of pregnancy. The remainder of the pregnancy was uncomplicated, with a negative prenatal congenital anomaly screening.

Due to progressive placental insufficiency and fetal growth restriction, cesarean section was performed at 32 weeks of gestation. The birth weight was 1450 g, with meconium‐stained amniotic fluid and an APGAR score of 6/8/8. During delivery, a maternal Group B *Streptococcus* (GBS) screening was positive, with massive colonization. The newborn was monitored in the neonatal intensive care unit with normal internal and neurological findings, required no rehabilitation, and was planned for discharge.

At 28 days of age, the infant experienced acute septic deterioration, with clinical findings dominated by neuroinfection symptoms, including irritability and seizure‐like activity. Blood cultures and throat swabs confirmed *S. agalactiae* (GBS). Intensive antibiotic therapy (amikacin, Prostaphylin, Taximed, and ampicillin) was initiated. Lumbar puncture revealed positive cytology, biochemistry, and polymerase chain reaction testing for GBS. After a week of antibiotic therapy, blood cultures became negative, neurological symptoms subsided, and EEG showed abnormal findings but no epileptic activity. Imaging studies, including cranial ultrasound, showed periventricular leukomalacia.

Before discharge, a newborn hearing screening was performed, revealing the presence of otoacoustic emissions (OAE) bilaterally but abnormal screening auditory brainstem response (ABR) results. A follow‐up was scheduled for 1 month later. At 3 months of age (corrected age: 1 month), OAE became absent, and ABR testing indicated mild hearing loss in the right ear and pathological findings in the left ear. Subsequent audiological testing showed progressive worsening, and the child was referred as a candidate for bilateral cochlear implantation.

**FIGURE 1:**
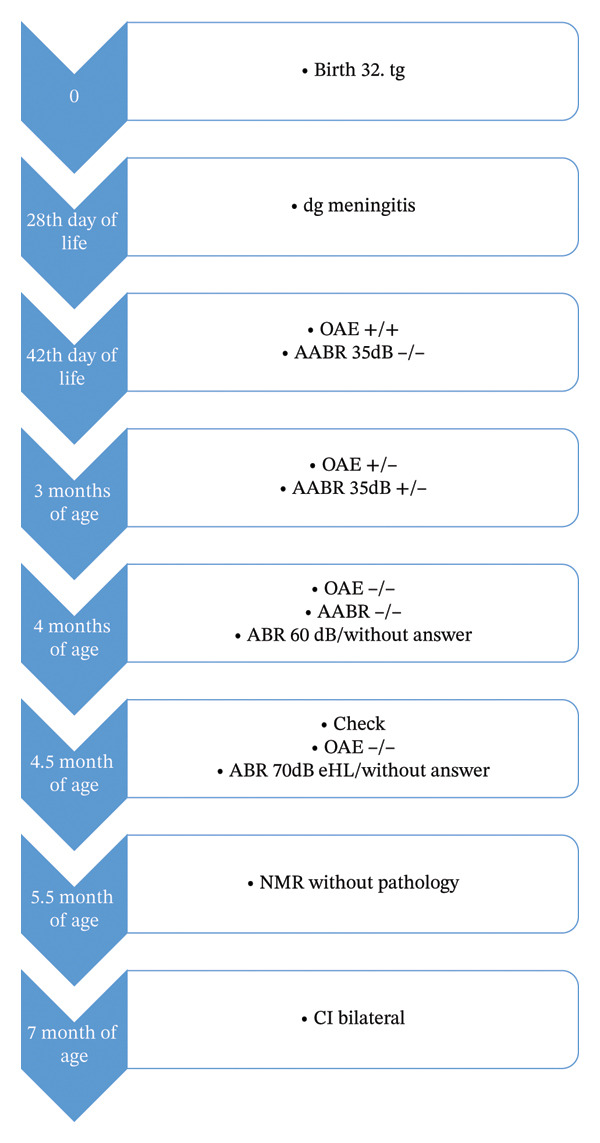
Timeline of the disease (diagnostics and treatment).

Preimplantation MRI at 5 months of age showed no abnormalities in the inner ear, with no signs of fibrosis or ossification. The cochlear nerves were bilaterally present and of normal appearance. Brain MRI, however, revealed multiple postischemic and postmalatotic pseudocysts, significant cortical involvement, and changes in the visual cortex. At 7 months of age (corrected: 5 months) and a weight of 7000 g, bilateral cochlear implantation was performed. During surgery, cochlear drilling was necessary due to significant ossification around the cochlear windows, also evident in the basal turn of the cochlea. The electrode array (MedEl Flex 28) was fully inserted without complications (Figure 1 shows the timeline of diagnostics and therapy). The speech processors were activated 2 months after surgery.

At the same time, the child was diagnosed with severe central visual impairment and moderate psychomotor developmental delay, but EEG showed no epileptic activity. Despite these severe consequences, significant improvements in auditory perception have been observed. Due to the visual impairment, a tactile sign language system is being developed on the body.

**FIGURE 2:**
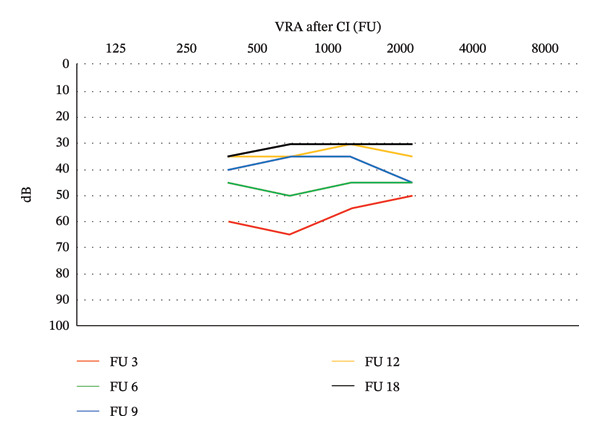
Visual reinforcement audiometry results after cochlear implantation.

Six months after cochlear implantation (hearing age: 4 months, gestational age: 13 months), the child’s global hearing capacity is at 45 dB in speech frequencies (Figure 2). Clear reactions to the mother’s voice and changes in intonation are observed. After activating the processor, the child calms down, stops crying, and repeats vowel sounds “A” and “U” (IT‐MAIS first milestone). At a hearing age of 7 months (gestational age: 16 months), the child responds reciprocally with motor actions to nursery rhymes (CAP 5).

### 2.2. Case 2

A 2.5‐year‐old boy was first examined at our clinic at 3 months of age following bacterial meningitis. He had been born prematurely at 34 weeks of gestation via cesarean section due to placental insufficiency. The amniotic fluid was clear, the APGAR score was 4/7/9, and the birth weight was 1700 g. The infant was initially stable in an incubator.

**FIGURE 3:**
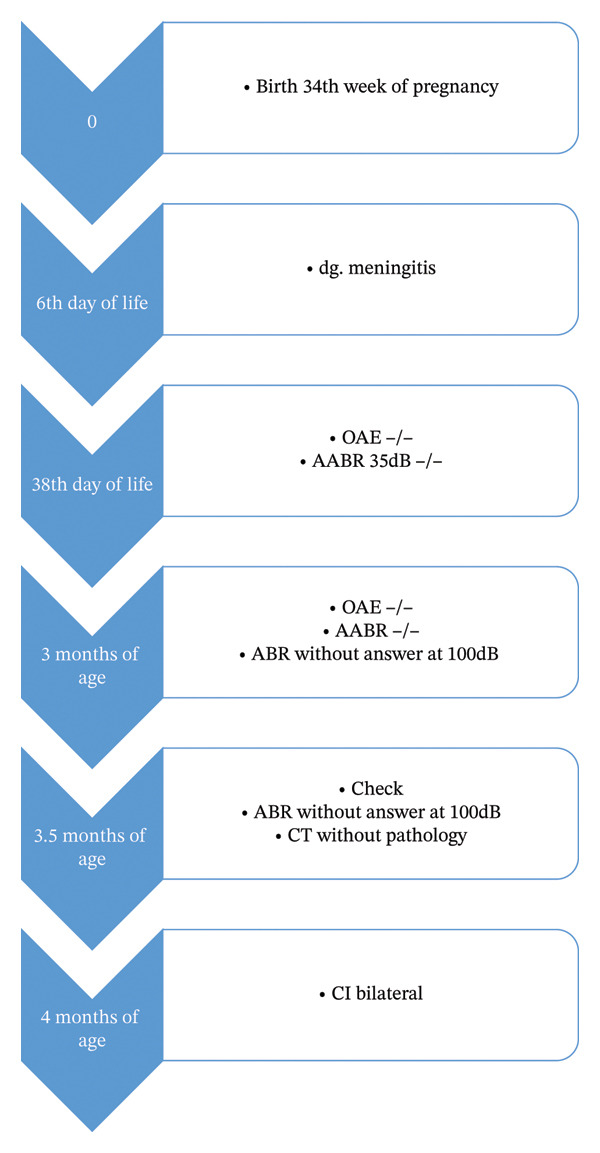
Timeline of the disease (diagnostics and treatment).

At 6 days of age, he developed sepsis, with *Serratia marcescens* confirmed in blood cultures. Clinically, he presented with fever and irritability. Lumbar puncture showed biochemical signs of meningitis but no microbiological findings. He required mechanical ventilation for 6 days and was treated with antibiotics (amikacin, ceftazidime, and meropenem). At 6 weeks of age, newborn hearing screening was performed, showing an absence of OAE and no response on ABR. He was referred to our center, where the diagnosis of profound hearing loss was confirmed. MRI showed no abnormality of the inner ear, and cochlear nerves were present bilaterally. CT demonstrated a cochlea without clear pathological changes or signs of ossification. At 4 months of age (corrected: 3 months) and weight 4000 g, bilateral cochlear implantation was performed. Electrode insertion was made through the round window, with full insertion of the MedEl Flex 28 array without complications (Figure 3 shows timeline of diagnostics and therapy). The speech processors were activated 3 months after surgery.

**FIGURE 4:**
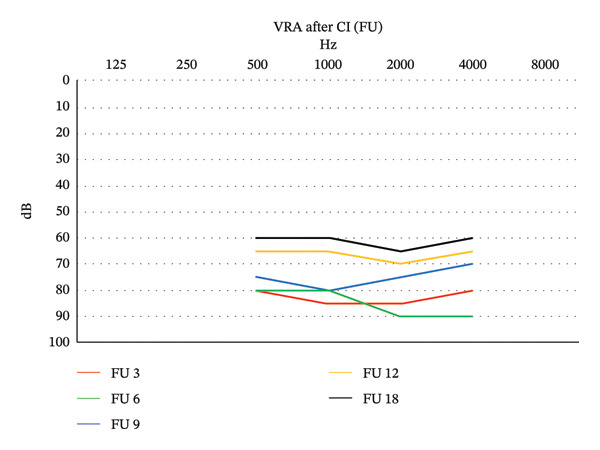
Visual reinforcement audiometry results after cochlear implantation.

At 6 months of age (corrected: 5 months), epileptic spasms developed, and EEG confirmed West syndrome. Severe psychomotor retardation persists to this day. Currently, the child has a hearing age of 24 months. His best global hearing capacity is approximately 60 dB HL, with reported responses to loud sounds in the home environment (Figure 4). According to parental observations, he vocalizes more while using the cochlear implants (CAP 1). To verify auditory pathway function, electrically evoked ABR (eABR) testing was performed via the cochlear implant, showing normal bilateral responses. Stimulation at 200 current units (CU) at electrodes 1 and 6 elicited a physiological wave II–V complex on both sides. Despite these favorable objective findings, functional auditory benefit remains limited (CAP 1). The child removes the speech processors for most of the day and reportedly responds only to general environmental sounds. These findings suggest that the poor functional outcome is more likely related to severe neurological comorbidity than to insufficient cochlear implant stimulation itself.

## 3. Discussion

Neonatal meningitis is a severe infectious disease with a significant risk of neurological complications. Thanks to high standards in intensive care units and effective antimicrobial therapy, mortality rates have declined, but morbidity remains high. The immature immune system and high permeability of the blood–brain barrier pose significant risk, particularly in newborns and especially in those born prematurely.

The estimated incidence in developed countries is 0.3 per 1000 newborns. In developing countries, it ranges from 0.8 to 6 per 1000 newborns. *S. agalactiae* remains the primary pathogen despite antibiotic prophylaxis in pregnant women. Among preterm infants, *E. coli* is the dominant pathogen [1–4].

Infection symptoms such as fever, irritability, or, conversely, increased lethargy appear very early after birth. Infections occurring within the first 72 h are classified as early‐onset infections, while those appearing later are termed late‐onset infections. Early‐onset meningitis is often associated with *S. agalactiae*, which the infant acquires during passage through the birth canal of an infected mother or, in some cases, through intrauterine ascending infection from the maternal genitourinary system [4, 5].

In countries with established prenatal screening programs and timely intrapartum prophylaxis for positive mothers, the incidence of early‐onset neonatal sepsis and meningitis has declined. Late‐onset meningitis is often caused by bacterial transmission to the newborn’s immature immune system from the surrounding environment, such as infected breast milk or nosocomial transmission [5].

There are many complications associated with bacterial meningitis. The first signs of infection can include irritability, seizures, and focal neurological deficits, while serious neurological complications often develop later, frequently after therapy. Among these complications are hearing loss, epilepsy, visual impairment, cognitive impairment, and delays in psychomotor development [2].

Sensorineural hearing loss is a well‐reported neurological consequence of bacterial meningitis, especially in adults and children, but it is less commonly seen in newborns. The primary causes of sensorineural hearing loss after bacterial meningitis are *Neisseria meningitidis* and *Streptococcus pneumoniae*. These types of meningitis typically occur in late childhood, while isolated *S. agalactiae* and other pathogens are much rarer [6].

The cochlea is the main site of impairment. The infection can enter the cochlea in several ways. The most common route is the direct spread of bacterial products from the subarachnoid space to the perilymphatic space of the cochlea and the labyrinthine system through the cochlear aqueduct. Another possible route is through sepsis or via the inner ear canal and the auditory nerve. At the cochlear level, damage occurs to the hair cells, supporting cells, and stria vascularis, as well as through degeneration of the spiral ganglion cells. Cochlear inflammation has been shown to cause fibrosis, which subsequently leads to cochlear ossification, typically developing 3–4 weeks after the onset of meningitis. Ossification begins in the basal turn of the cochlea, near the round window, where the cochlear aqueduct enters the cochlea. This leads to a high risk of not being able to implant cochlear electrodes [4, 7].

Various authors report different times between the onset of meningitis and hearing impairment. It is believed that hearing loss occurs early in the illness and progresses rapidly, typically within 48 h after the onset of the disease [4, 8]. In our cases, at the time of discharge to the home environment, which was 3 weeks after onset of the illness, OAEs were present. The deterioration occurred more than a month after the onset of the disease. Hearing impairment also can result from damage to the auditory nerve or a central auditory lesion [4, 8]. Severe damage to the spiral ganglion cells can lead to poorer outcomes after cochlear implantation compared to cases of purely cochlear damage. Due to the early onset of cochlear ossification, cochlear implantation must be performed as soon as possible. In neonatal meningitis, the disease’s course is often very dramatic, with a need for mechanical ventilation. For these reasons, the diagnosis of hearing impairment is often delayed until the patient is stabilized, sometimes several weeks after onset of the disease. Additionally, the young age of the patient often makes early detection of hearing loss difficult [6]. Diagnostic methods should rely on objective tests, including OAE and brainstem auditory evoked potentials. Although the literature suggests that cochlear injury may occur very early in the course of meningitis, our observations indicate that hearing deterioration may not always be fully apparent during the acute phase. In Case 1, OAEs were initially present but later disappeared while hearing progressively worsened. This finding supports the need for repeated audiological monitoring even after apparent clinical stabilization.

Several studies suggest the suitability of using dexamethasone to reduce cochlear ossification and alleviate hearing impairment, but only if it is administered concurrently with antibiotic therapy at onset of the disease. Some studies report reduction in the side effects after administering dexamethasone in meningitis caused by *Haemophilus influenzae*, but its effectiveness has not been proven for *S*. *pneumoniae*. Adjunctive dexamethasone in the treatment of acute bacterial meningitis does not seem significantly to reduce death or neurological damage [9, 10]. Neither child received adjunctive dexamethasone during the acute phase of meningitis because this was not part of the treatment protocol at the referring neonatal unit at that time. Information about the status of the inner ear, fibrosis, and cochlear ossification can be obtained using imaging methods. High‐resolution CT is particularly useful in more advanced stages of ossification, whereas MRI may detect earlier inflammatory or fibrotic changes. Contrast‐enhanced T1‐weighted MRI with gadolinium has been reported to demonstrate acute labyrinthitis through enhancement related to increased vascular permeability, while T2‐weighted sequences may show reduced cochlear fluid signal in the presence of fibrosis. In our first case, preoperative MRI was performed without gadolinium contrast enhancement, which may have limited the sensitivity for detecting active inflammatory changes. Although this examination at 5 months of age showed no signs of fibrosis or ossification, implantation performed 2 months later required drilling because of marked ossification around the cochlear windows and within the basal turn. This suggests that MRI may underestimate early or evolving postinflammatory cochlear changes in some infants after meningitis. Therefore, negative imaging findings should not delay candidacy assessment or timely cochlear implantation when profound postmeningitic hearing loss is suspected [6, 7, 11].

The implantation process itself is challenging for the surgeon in infants, both due to anatomical considerations and because of the small blood volume, making thorough hemostasis crucial. Due to potential cochlear ossification, a standard approach is often not possible, and an alternative method must be chosen. Depending on the degree of ossification, the electrode can be placed via cochleostomy, by “drilling” through the newly formed bone of the basal turn, or by placing the electrode into the scala vestibuli. In our patient, access through the round window was not possible due to ossification in this area, so the approach via cochleostomy with full electrode insertion was chosen. The course of the operation was without complications [12].

The outcome of cochlear implantation and its effect on speech comprehension and development are influenced by a variety of factors. Some patients achieve very good results, while others are only able to perceive ambient sounds. An important factor is the degree of cochlear ossification and depth of electrode insertion. Because damage to the inner ear is not limited to the cochlea and may also affect the dendrites of the spiral ganglion, insufficient electrical stimulation may occur even with full insertion [4, 7]. In such cases, the outcome of implantation can be verified using eABR. In the case of the boy, where there was insufficient effect after cochlear implantation, normal function of the auditory pathway was confirmed using eABR. Equally important is the delayed cognitive and neural development of patients following meningitis. In our cases, and despite full electrode insertion in both children, the effect of cochlear implantation in the first patient is limited by visual impairment and that in the boy by severe developmental delay.

## 4. Conclusion

The diagnosis of profound hearing loss and cochlear implantation in infants who are deaf due to bacterial meningitis is a challenge for everyone involved. Determining the severity of the hearing loss is often delayed until the patient’s condition is stabilized. Due to cochlear ossification, early cochlear implantation is required. Very young age and low weight of the patient do not present an obstacle to performing a successful implantation, as proven by the cases we have presented here. The outcome is often influenced by other neurological complications. Very young age and low body weight do not necessarily preclude successful cochlear implantation, provided that the procedure is performed in a timely manner in an experienced center.

## Author Contributions

Dagmar Hošnová, Milan Urík, and Vít Kruntorád: conceptualization, manuscript preparation, data acquisition, and data analysis. Jan Šíma: data acquisition, data analysis, and language revision. Milan Urík: supervision and revision of the manuscript.

## Funding

This work was supported by the Ministry of Health of Czech Republic, Conceptual Development of Research Organisation (FNBr, 65269705), and Masaryk University in Brno (MUNI/A/1543/2024).

## Ethics Statement

Written informed consent from the patient’s legally authorized representatives for the publication was obtained.

## Conflicts of Interest

The authors declare no conflicts of interest.

## Data Availability

All data are available from corresponding author.
